# A New Set of Chemical Starting Points with *Plasmodium falciparum* Transmission-Blocking Potential for Antimalarial Drug Discovery

**DOI:** 10.1371/journal.pone.0135139

**Published:** 2015-08-28

**Authors:** Maria Jesus Almela, Sonia Lozano, Joël Lelièvre, Gonzalo Colmenarejo, José Miguel Coterón, Janneth Rodrigues, Carolina Gonzalez, Esperanza Herreros

**Affiliations:** 1 GlaxoSmithKline, Tres Cantos Medicines Development Campus-Diseases of the Developing World, Severo Ochoa 2, Tres Cantos, 28760, Madrid, Spain; 2 Universidad Nacional de Educación a Distancia (UNED), C/ Senda del Rey 11, 28040, Madrid, Spain; 3 GlaxoSmithKline, Basic Research Center, Computational Chemistry Department, C/ Santiago Grisolía 4, Tres Cantos, 28760, Madrid, Spain; Bernhard Nocht Institute for Tropical Medicine, GERMANY

## Abstract

The discovery of new antimalarials with transmission blocking activity remains a key issue in efforts to control malaria and eventually eradicate the disease. Recently, high-throughput screening (HTS) assays have been successfully applied to *Plasmodium falciparum* asexual stages to screen millions of compounds, with the identification of thousands of new active molecules, some of which are already in clinical phases. The same approach has now been applied to identify compounds that are active against *P*. *falciparum* gametocytes, the parasite stage responsible for transmission. This study reports screening results for the Tres Cantos Antimalarial Set (TCAMS), of approximately 13,533 molecules, against *P*. *falciparum* stage V gametocytes. Secondary confirmation and cytotoxicity assays led to the identification of 98 selective molecules with dual activity against gametocytes and asexual stages. Hit compounds were chemically clustered and analyzed for appropriate physicochemical properties. The TCAMS chemical space around the prioritized hits was also studied. A selection of hit compounds was assessed *ex vivo* in the standard membrane feeding assay and demonstrated complete block in transmission. As a result of this effort, new chemical structures not connected to previously described antimalarials have been identified. This new set of compounds may serve as starting points for future drug discovery programs as well as tool compounds for identifying new modes of action involved in malaria transmission.

## Introduction

The last decade has witnessed unprecedented progress in reducing the incidence of *Plasmodium falciparum* malaria, especially in Africa where the burden of disease is greatest. Continuous efforts to reduce malaria cases, such as insecticide treated nets, indoor residual spraying and artemisinin-based combination therapies (ACTs) have contributed to a significant decrease in malaria mortality, from an estimated 1,800,000 deaths in 2005 to 584,000 registered in 2014 [1]. Nevertheless, in order to achieve the ultimate goal of eradicating malaria, more effective tools will be required. These include efficacious vaccines and innovative antimalarial drugs with novel mechanisms of action displaying not only efficacy against resistant parasites but also transmission blocking potential.

The World Health Organization (WHO) currently recommends ACTs as the standard of care for uncomplicated malaria in endemic countries. However, since 2008 [2], artemisinin resistance has been described in western Cambodia, Thailand and Vietnam, threatening malaria control, treatment and elimination efforts worldwide [3]. In order to block transmission, ACTs must be combined with primaquine, the only drug recommended by the WHO at single low dose for this indication. However, primaquine safety is of particular concern given its hemolytic side effects in patients with glucose-6-phospate dehydrogenase (G6PD) deficiency. Consequently, there is an urgent need to develop alternative antimalarial drugs to treat acute malaria in individual patients and prevent the spread of infection in the population.

During the past decade, phenotypic screenings have focused on identifying compounds with activity against *P*. *falciparum* asexual blood stages, responsible for proliferation of the parasite in the human host and for the clinical symptoms of malaria. Millions of compounds have been tested and thousands of new molecules identified, some of which are currently undergoing development [4, 5]. As part of this effort, GlaxoSmithKline published the Tres Cantos Antimalarial Set (TCAMS), comprising 13,533 compounds resulting from the screening of nearly two million compounds from the GSK corporate collection [6]. The TCAMS set described high quality starting points demonstrating potent activity against malaria asexual stages and physicochemical traits suitable for the discovery of orally administrated drugs. Consequently, a number of new oral antimalarial agents are now under investigation [7].

The next challenge in malaria drug development is to identify molecules with activity against mature gametocytes, with consequent transmission blocking potential. Gametocytes are the parasite stage responsible for transmission of the disease through the *Anopheline* vectors [8]. In the case of *P*. *falciparum* malaria, gametocytes develop from committed schizonts over a period of 12 days [9]. Successive differentiation events lead to early gametocytes (stages I to III), susceptible to most of classical schizonticidal antimalarials. These finally develop into mature gametocytes (stages IV and V) which are insensitive to most commercial antimalarial drugs. Only the fully developed mature stage V gametocytes can infect *Anopheles* mosquitoes.

A drug discovery strategy to identify a new class of antimalarial agents with dual activity against both the asexual and gametocyte parasite stages is highly desirable. Consequently, a new assay to screen compounds for their potential to inhibit stage V gametocytes was developed, validated against standard antimalarials, and included in our test cascades [10]. This *in vitro* assay measures ATP intracellular levels as a surrogate of gametocyte viability. This robust and reliable assay was adapted to the high-throughput screening (HTS) format.

Here we describe the screening of the TCAMS compound set for activity against *P*. *falciparum* stage V gametocytes. To our knowledge, this is the largest screening campaign conducted using *P*. *falciparum* sexual stages. Hundreds of primary hits with activity against both blood stages and gametocytes were identified and analyzed, applying sequential and progressively more demanding chemical, biological and developability filters. Finally, selected compounds were evaluated for their impact on transmission reduction to the *Anopheles stephensi* mosquito vector using the standard membrane feeding assay (SMFA), and thereby validating the integrated approach presented in this work.

## Results

### Phenotypic screening against mature gametocytes

**Fig 1:**
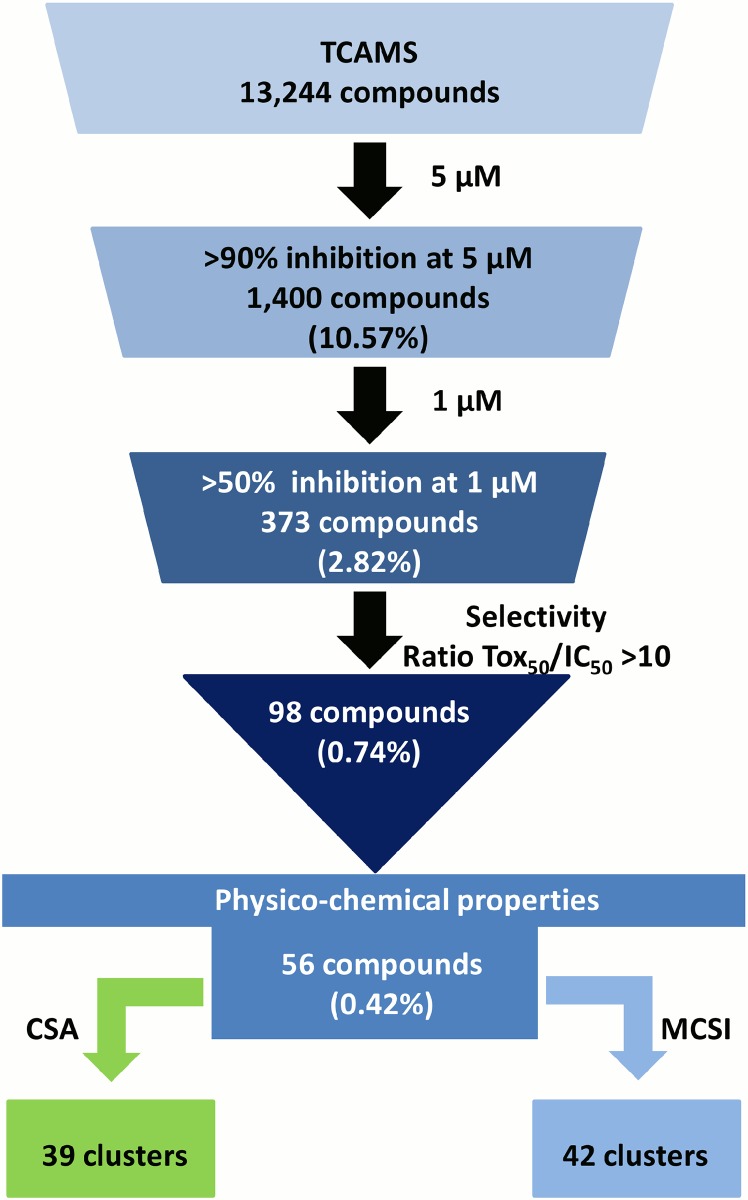
Description of the critical path followed to generate and prioritize hits from TCAMS.

The research protocol for this study is described in Fig 1. Briefly, the TCAMS compound set was screened using a bioluminescence ATP assay [10] to identify molecules with dual activity against *P*. *falciparum* asexual forms and stage V gametocytes. Subsequently, the primary hits were analyzed by applying a cascade of sequential and increasingly more demanding biological and physicochemical filters.

The initial screen of the entire TCAMS set identified 1400 compounds (10.57% hit rate) as positive hits for inhibition of mature gametocytes, based on a decrease in the level of ATP content by ≥ 90% at a single assay concentration of 5 μM. A summary of the HTS results is provided as S1 Table. The 1400 primary hits were tested in a second screen at a single concentration of 1 μM. A total of 373 compounds (2.82% hit rate) were identified that decreased ATP levels by ≥ 50% (S2 Table). An interference assay, using a panel of 167 compounds tested at 50 μM, showed no significant inhibition confirming that the assay had identified compounds with activity against mature gametocytes, rather than the BacTiter-Glo reaction itself (S3 Table).

**Fig 2:**
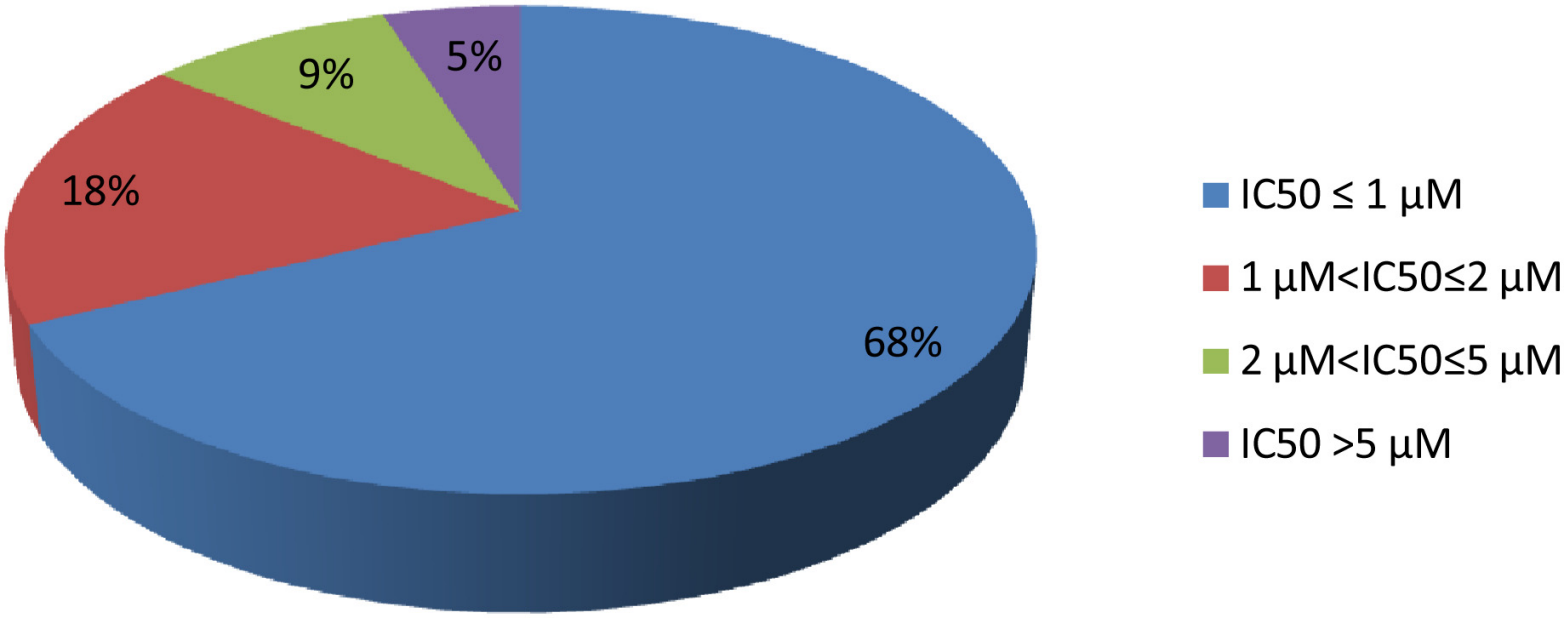
Relative distribution of TCAMS primary hits based on *P*. *falciparum* IC_50_ in gametocytes.

The 373 selected compounds were subsequently re-tested in a dose–response assay to determine IC_50_ values, with an average Z-prime factor of 0.55. There was excellent correlation between percent inhibition at 1 μM and the IC_50_ results. This suggests high reliability of the single point assays. As can be seen in Fig 2, nearly 70% of the hits displayed potencies ≤ 1 μM. However, there was no significant correlation between compound IC_50_ values obtained against gametocytes and the inhibition of growth of *P*. *falciparum* asexual forms as previously determined for TCAMS [6] (S4 Table).

In order to assess the antimalarial specificity of the 373 active compounds, their potential cytotoxicity was tested against HepG2 cells after 48 h of exposure using the same biomarker as for the gametocyte assays (depletion of intracellular ATP levels). Compounds were selected with potencies of < 2 μM from the dose–response curves in *P*. *falciparum* and a selectivity ratio > 10 in the cytotoxicity test (i.e. activity in the human hepatoma cell line divided by activity in gametocytes > 10). As a result, 98 compounds were progressed for subsequent analysis (S5 Table).

### Chemical filtering, analog search and selection of drug-like molecules

**Fig 3:**
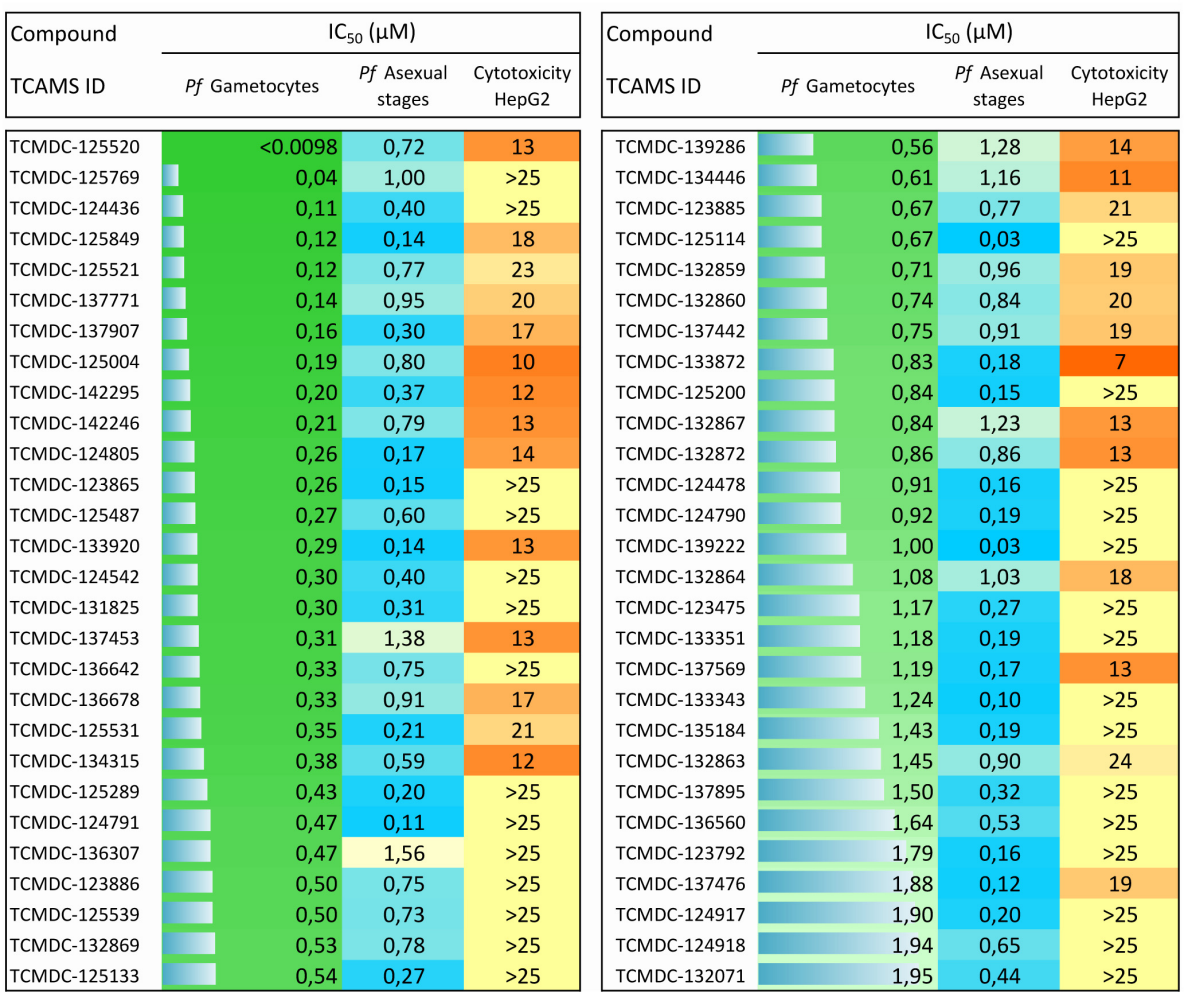
Biological profile of the 56 prioritized compounds. Bars and color intensity represent compound potency against *P*. *falciparum* gametocytes asexual stages, and cytotoxicity.

**Fig 4:**
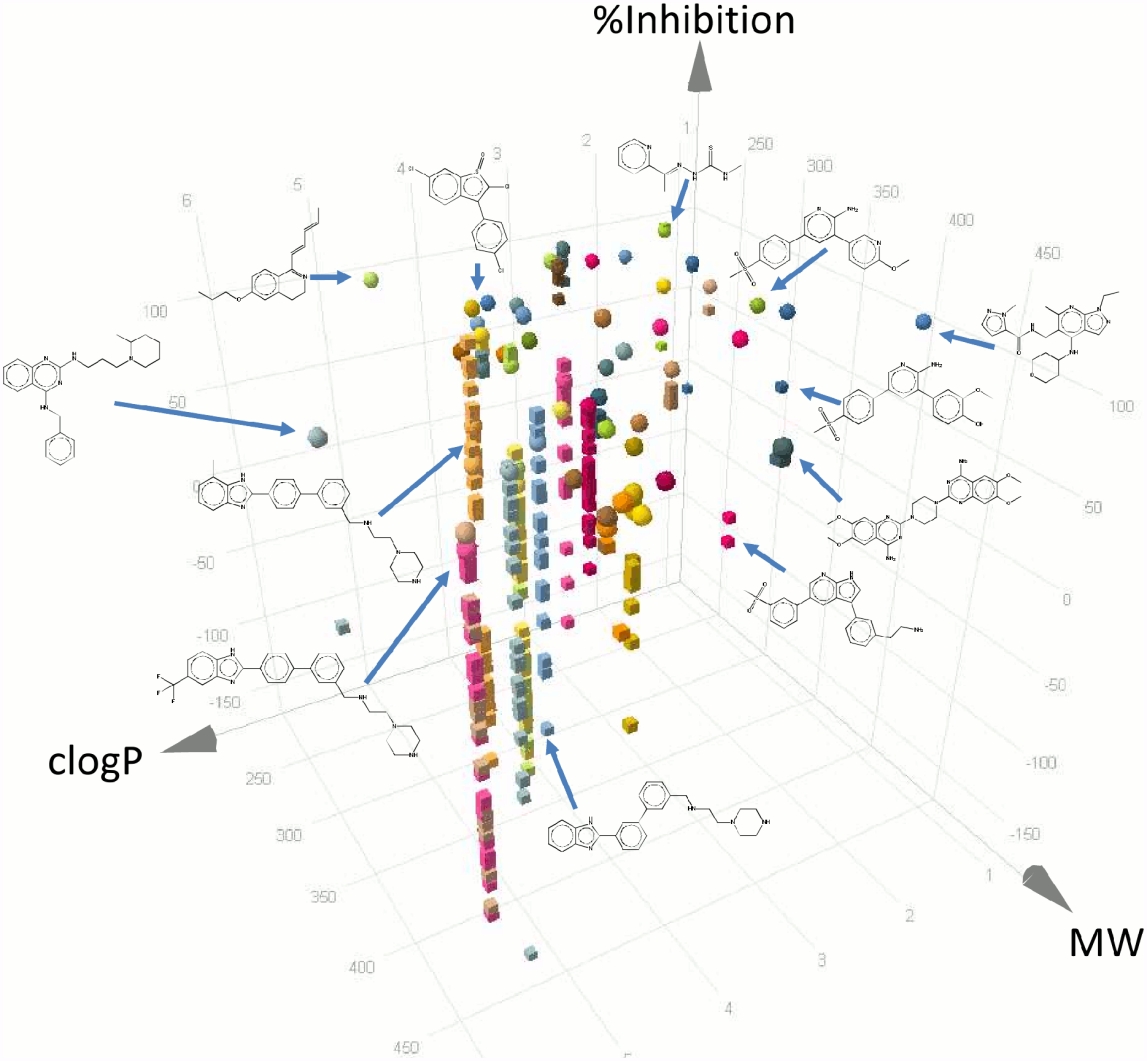
Three-dimensional plot displaying the structures, properties and parasitological activity of some of the 56 prioritized compounds and their analogs. Selected compounds are represented as spheres, while the corresponding analogs (displayed at the same clogP and molecular weight as their proposed compound, but with their own inhibition value) are represented as cubes.

Hits were further narrowed down based on physicochemical parameters, attempting to select the most developable assets. Cutoffs, such as molecular weight ≤ 500, clogP ≤ 5, number of aromatic rings ≤ 4, number of rotatable bonds ≤ 10, number of hydrogen bond acceptors ≤ 10, and number of hydrogen bond donors ≤ 5, were used (Fig 1). A full description of the physicochemical properties of the 98 compounds is provided (S6 Table). This process led to prioritization of 56 drug-like compounds (S7 Table). Their biological activity and physicochemical properties are summarized in Figs 3 and 4.

Sorting similar structures into clusters is a valuable tool in the drug discovery process. Two different approaches were applied to analyze the TCAMS chemical space around the 56 prioritized compounds.

1. Similarity-based computational search of analogs (CSA) identified 25 clusters and 14 singletons (Fig 5a, Fig 5b and S8 Table), and retrieved 434 analogs associated to the different clusters (S9 Table).

**Fig 5a:**
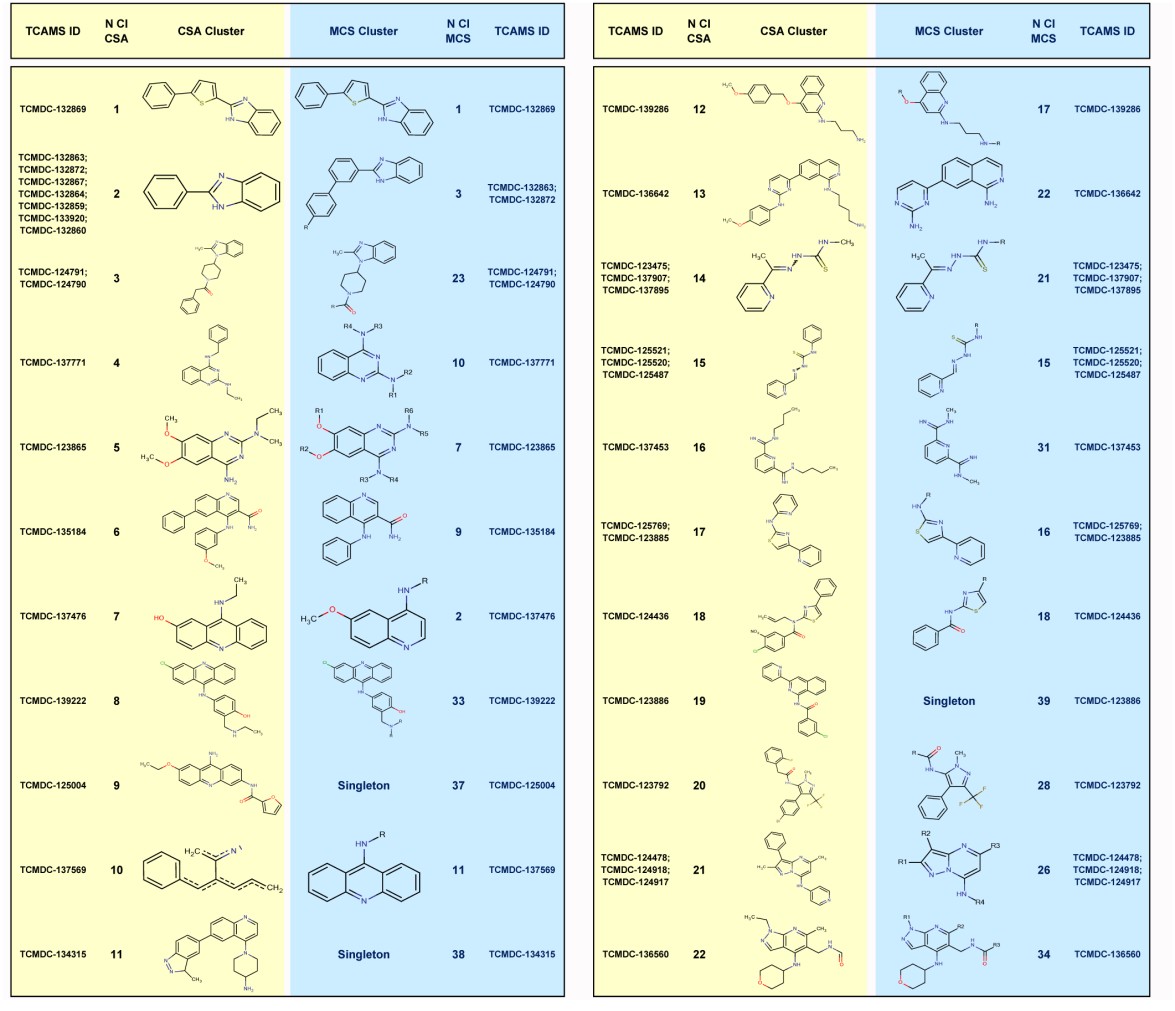
Chemical analysis of the 56 prioritized hits by CSA and MCSI methods. "TCAMS ID" represents the reference of the hit compound at TCAMS. "CSA Cluster" refers to the chemical scaffold obtained using the similarity-based computational search of analogs approach. "N Cl CSA" is the number of cluster using CSA approach. "MCS Cluster" depicts the chemical scaffold obtained using the similarity-based medicinal chemistry search of analogs approach and "N Cl MCS" indicates the number of cluster using MCS approach.

**Fig 5b:**
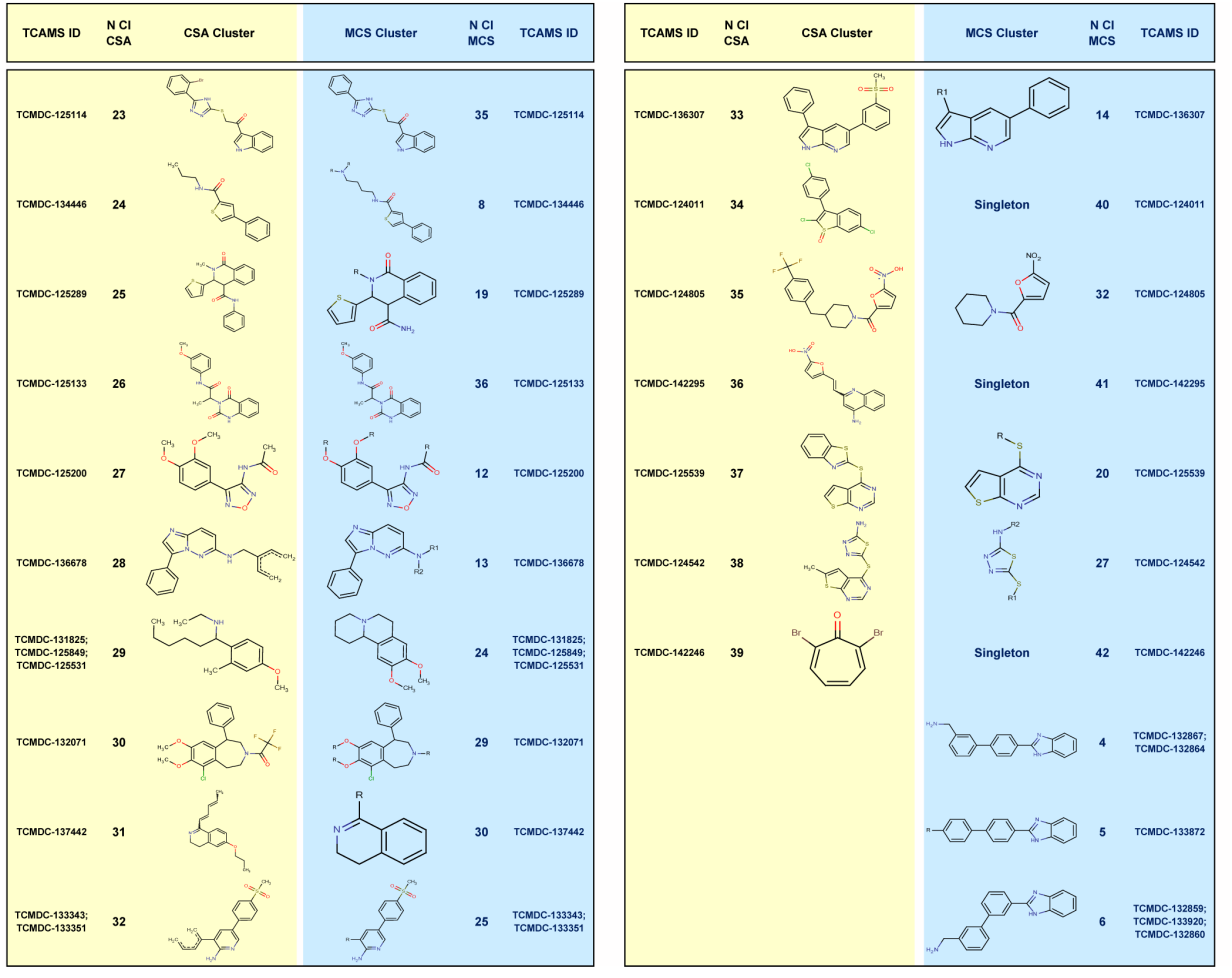
Chemical analysis of the 56 prioritized hits by CSA and MCSI methods. "TCAMS ID" represents the reference of the hit compound at TCAMS. "CSA Cluster" refers to the chemical scaffold obtained using the similarity-based computational search of analogs approach. "N Cl CSA" is the number of cluster using CSA approach. "MCS Cluster" depicts the chemical scaffold obtained using the similarity-based medicinal chemistry search of analogs approach and "N Cl MCS" indicates the number of cluster using MCS approach.

2. Substructure-based medicinal chemistry scaffold indexing (MCSI) identified 36 clusters and 6 singletons (Fig 5a, Fig 5b and S8 Table), together with 1252 associated analogs within the TCAM set (S10 Table).

For each chemical cluster, the corresponding analogs presented different substitution patterns around the common chemical substructure, maximizing the probabilities of building structure–activity relationships for each chemical substructure.

### Confirmation of the transmission-blocking potential of selected hits in the SMFA

**Fig 6:**
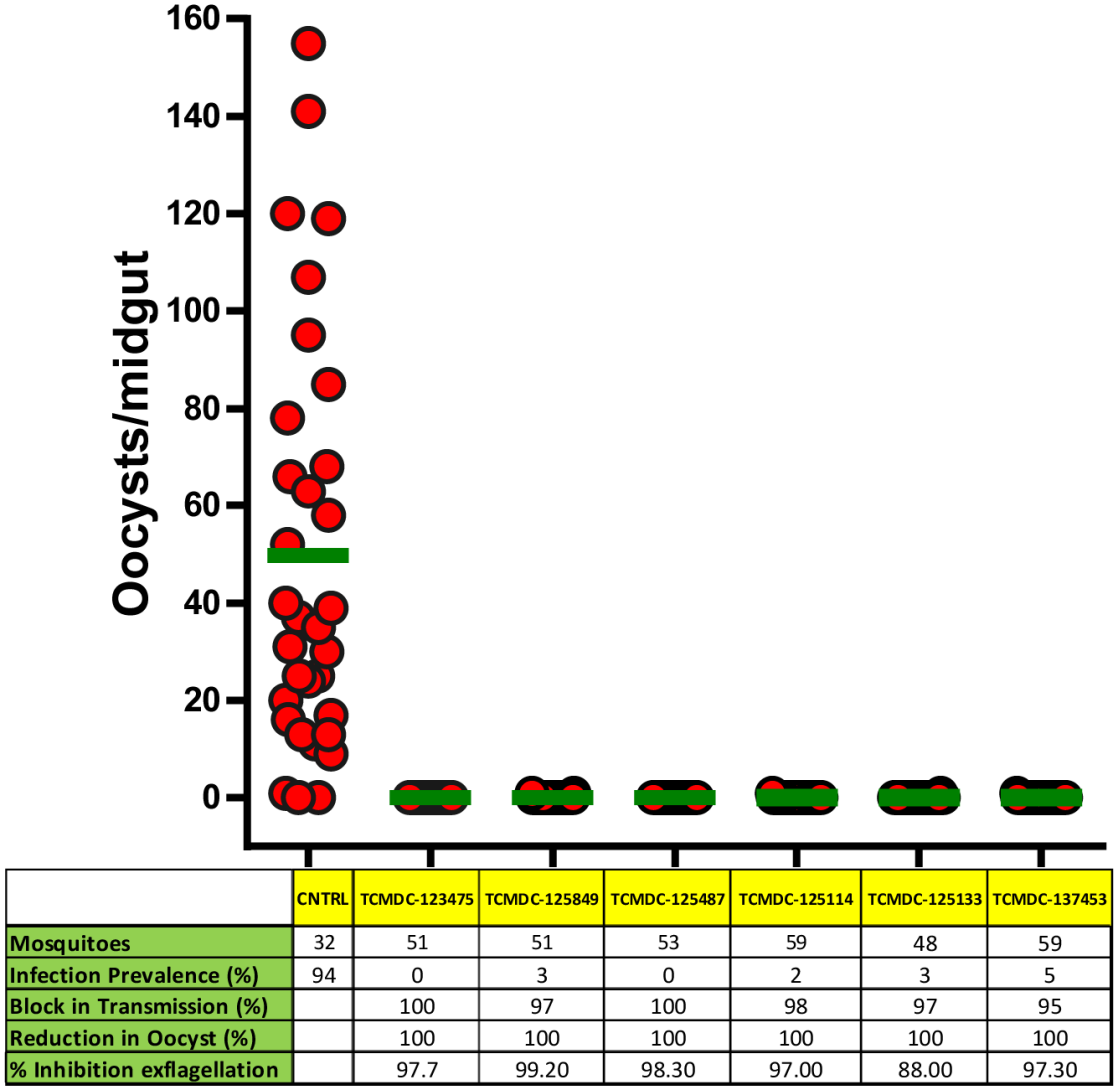
Effect of selected TCAMS on *P*. *falciparum* oocyst intensity after treatment of mature gametocytes for 48 h. In the figure, each red dot represents the total number of oocysts in a single mosquito midgut, and the green line depicts the mean oocyst intensity of infection. The table below the graph depicts the total number of full-fed mosquitoes dissected per treatment, the percentage infection prevalence, transmission blocking potential, mean oocyst intensity reduction, and the percentage inhibition of exflagellation at 48 h post-treatment. The percentage transmission blocking potential, mean oocyst intensity reduction and exflagellation inhibition were calculated by normalizing with values from the control group. Compounds showed a statistically significant decrease in oocyst distribution when compared to the control (p<0.0001). The data above are representative from one of the two independent experiments.

The *in vitro* gametocytocidal activity was validated *ex vivo* using the SMFA to test the efficiency of a selection of molecules in preventing the infection of mosquitoes. Six molecules displaying a wide range of anti-gametocyte activities varying from 0.12 to 1.17 μM, and representing great structural diversity within the drug-like space, were selected for evaluation in *Anopheles stephensi* mosquitoes. The transmission blocking potential of these molecules was measured as the percentage reduction in the prevalence of infection and mean oocyst intensity compared to DMSO-treated controls. As a previous step, the capability of these compounds to inhibit male gametocyte exflagellation was also tested. Following exposure to a concentration equivalent to the IC_90_ value obtained in the ATP assay, all six compounds blocked exflagellation, (≥ 88% inhibition, Fig 6).

**Fig 7:**
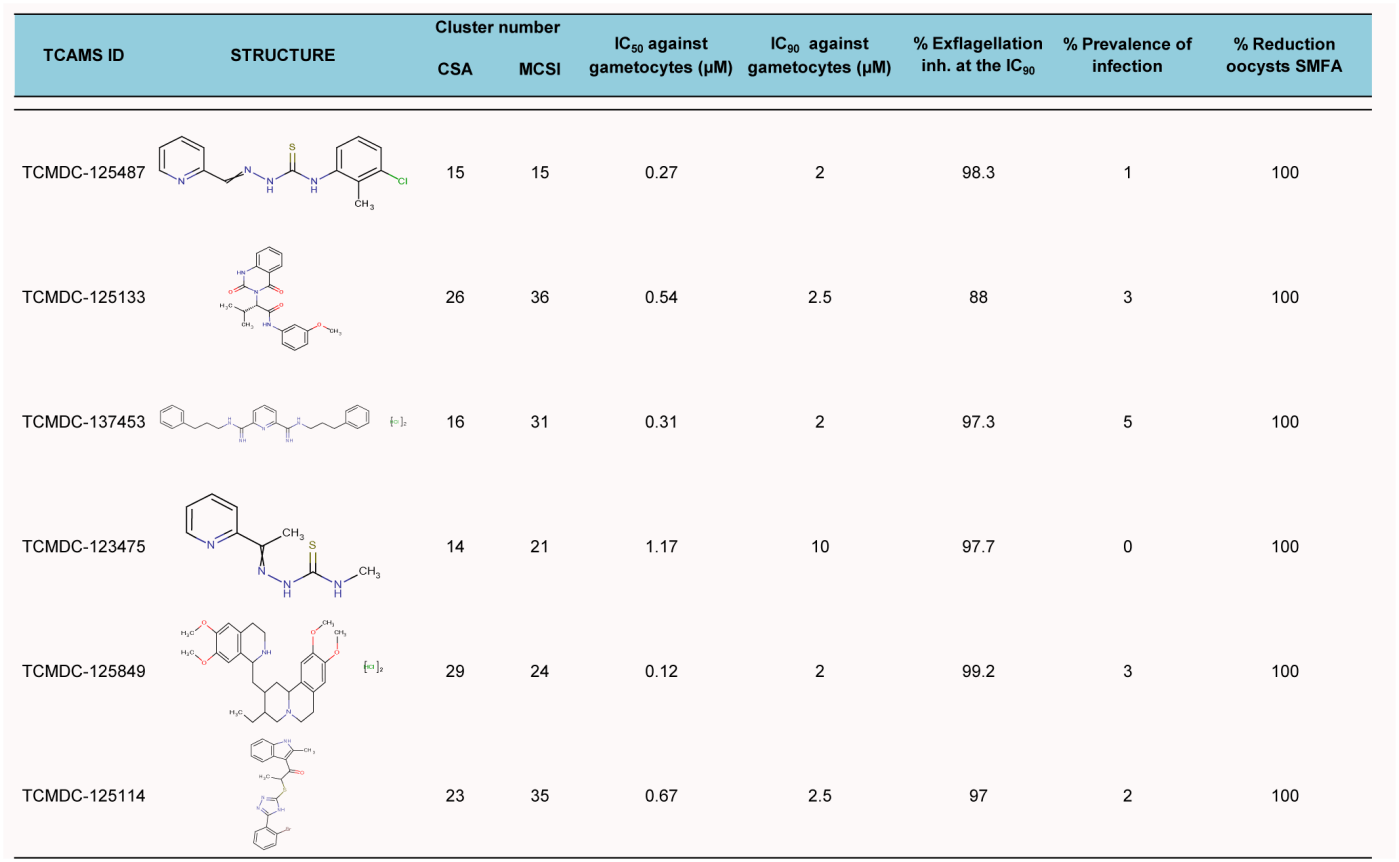
Summary of biological data for the 6 compounds evaluated in the SMFA.

Figs 6 and 7 show the ability of selected compounds to prevent *P*. *falciparum* transmission to mosquitoes. The prevalence of infection after gametocyte treatment ranged from 5% to 0%, which corresponds to a block in transmission from 95% to 100% (Fig 6). An infection prevalence of 94% was observed in control mosquitoes. The inhibition of mosquito infection showed an excellent correlation with the IC_90_ values obtained *in vitro* (Fig 7). All the compounds produced a 100% reduction in mean oocyst numbers.

## Discussion

Several extensive small molecule screens against *P*. *falciparum* blood stages have been published, expanding the portfolio of potential candidates for treating acute malaria. Nevertheless, there is still a crucial need for antimalarial drugs that go beyond treating acute infections, and which have the potential to clear circulating gametocytes, thus contributing to eradicate the disease.

The main objective of this work was to identify promising antimalarials from the 13,533 molecules within the GSK TCAMS library which, in addition to demonstrated *in vitro* activity against asexual stages, could also display transmission blocking potential. Furthermore, to find drug-like molecules, screening based on developable properties was applied. In accordance to these objectives, the biological screens were performed following increasingly stringent filters. This approach enabled us to establish a primary list of 373 compounds with potencies in the nanomolar range against both asexual and sexual stages of *P*. *falciparum*.

To conduct such extensive gametocyte screens, some issues need to be resolved. For example, large numbers of mature gametocytes have to be generated in a reproducible manner and a robust assay with a fast and simple read-out suitable for HTS developed. Although several methods for assessing antimalarial activity in gametocytes have been published and used to screen compound collections [11–16], few have been validated by testing mosquito infectivity.

The ATP-bioluminescence assay used in this work fulfills the requirements of HTS [10]. Previously, the assay has been shown to yield a signal proportional to the number of viable cells [17, 18]. The possibility of selecting false-positive hits, i.e., molecules inhibiting the bioluminescence reaction rather than killing gametocytes, was examined. None of the tested compounds proved to be an inhibitor of the luciferase reaction. Another result supporting the robustness of our ATP gametocytocidal assay is the high re-test rate obtained (approximately 70%). Furthermore, in this study the ATP-bioluminescence assay was further validated *ex vivo* in the SMFA, currently considered the gold standard for assessing molecules with transmission blocking potential. A test selection of six positive hits identified using the ATP-bioluminescence assay had exceptional activity in preventing the infection of *Anopheles stephensi* mosquitoes. These findings strongly support the predictive value of this methodology.

High hit rates were obtained on the HTS screening of TCAMS at 5 μM (10.57%) and at 1 μM (2.82%) compared with the typical range of values for random screenings (0.1–5%) [19]. The higher rates observed in this screen could be explained as the result of an 'enrichment’ of the antimalarial properties of the starting compound collection, as TCAMS is entirely composed of molecules active against asexual stages of *Plasmodium*.

In order to secure the early exclusion of potentially toxic molecules from the progression cascade, the 373 dual-acting compounds were tested in mammalian HepG2 cells, also using ATP depletion as readout methodology. The cytotoxicity assessment narrowed down the list of promising compounds to 98 molecules which displayed a selectivity ratio > 10. It is interesting that the percentage of compounds exhibiting inhibition > 50% at 10 μM in the HepG2 cell line was much lower in TCAMS overall (14.55%) than in the 373 dual-acting compounds (51.81%). This could be because mature gametocytes are non-dividing quiescent cells, while asexual stages are growing cells with an active metabolism. Compounds simultaneously targeting gametocytes and asexual stages could be affecting housekeeping processes also present in mammalian cells. This hypothesis suggests that it would be advisable to screen unbiased compound collections, not previously filtered by their activity in blood stages, for activity against gametocytes. Nevertheless, it is encouraging that 26.3% of the 373 dual-acting compounds displayed a selectivity ratio > 10, suggesting the existence of specific targets important for both asexual and sexual stages, but not present in mammalian cells.

There was no significant correlation between the potency of compounds in gametocytes compared with the activity in asexual forms [6]. A number of factors could explain this observation. Firstly, the inhibition data were obtained using quite different methodologies for each study (lactate dehydrogenase determination for asexual forms and ATP content for gametocytes). Another factor could be the low number of analogs composing the clusters studied, thus reducing statistical significance. There may also be differences in drug susceptibility between life-cycle stages or even different targets involved in asexual and sexual parasite stages.

Fifty-six molecules were prioritized in terms of drug-like and biological properties. The application of these filters does not guarantee success in identifying developable scaffolds, but aims to reduce the attrition risk in drug development. Two complementary chemoinformatic tools (CSA and MCSI) were applied to classify the selected molecules. While CSA was more efficient when the objective was hit expansion through the identification of active analogs, MCSI was the approach of choice for building structure–activity relationships around a common scaffold, which is very useful for a phenotype-based approach like TCAMS.

Hit structural analysis showed that few compounds included well-known single antimalarial scaffolds (quinacrine-like in TCMDC-137476 and TCMDC-137569) and double antimalarial sub-structures (amodiaquine-like + quinacrine-like in TCMDC-139222). Some other chemotypes described recently were also perceived (SAHH2-like in TCMDC-123865 [20]; MMV390048-like in TCMDC-133343 and TCMDC-133351 [21]; compound **13**-like in TCMDC-125539 [22]; compound C2-1-like in TCMDC-123792 [23]; and SJ733-like in TCMDC-125289 [24]. However, most of the 56 selected molecules were new structures not connected to either a classical or recently described antimalarial scaffolds.

TCAMS is annotated with target information for each compound, if known. Annotations for the 56 compounds include two (TCMDC-123885 and TCMDC-125769) which have been reported as inhibitors of KCa2.2, a calcium-dependent K^+^ channel. Interestingly, recent bioinformatic analyses suggest the presence of homologues of calcium-activated K^+^ channels in the *P*. *falciparum* genome [25]. The only other compound with a target annotation is TCMDC-123886, reported as an adenosine A3 receptor ligand. The rationalization for this target is more problematic as there is little evidence for G-protein-coupled receptors (GPCRs) in *P*. *falciparum*. However, GCPRs are of ancient origin and present in almost all eukaryotic organisms so far examined [26]. Thus, further research is required to validate or exclude adenosine A3 as a target in *P*. *falciparum*. In addition, both TCMDC-123792 and TCMDC-125289 correspond to chemotypes that have been characterized as inhibitors of a P-type cation-ATPase (PfATP4) [23, 24]. The remaining 51 prioritized compounds lack target annotation in TCAMS. By publishing these novel structures and providing the associated data as open access, we hope to stimulate research into identifying validated targets for drugs acting against *P*. *falciparum* gametocytes.

The Malaria Box is a collection of 400 diverse compounds with confirmed activity against *P*. *falciparum* asexual forms [27]. This set has been assembled by Medicines for Malaria Venture (MMV) in an attempt to catalyze drug discovery and research in malaria and other neglected diseases. The collection is composed of molecules from St. Jude Children's Research Hospital, Novartis-GNF, commercial libraries, and GSK (140 molecules belonging to TCAMS). This set has been screened against mature gametocytes by other groups [12, 14–16], presenting a low degree of coincidence among them. Gathering the published hit lists, a total of 53 compounds from TCAMS were identified. When compared with our prioritized molecules, 15 structures were found to be in common. No more coincidences were found with those other screens, indicating a high degree of chemical novelty for the set of hits presented in this work.

## Conclusion

Screening one of the largest antimalarial compound collections (TCAMS) identified molecules with activity against both *P*. *falciparum* asexual and gametocyte stages. The set of selected compounds with transmission-blocking potential includes well-known scaffolds as well as promising new chemical entities, showing a high degree of chemical novelty. Further parasitological, toxicological and pharmacokinetic studies will be required to identify antimalarial leads and pre-clinical candidates. Most importantly, the results suggest new starting points for malaria drug discovery and have been provided open access to enable further research into the prevention of malaria transmission.

## Materials and Methods

### Ethics statement

Human biological samples were ethically sourced, and their research use was in accordance with the terms of the informed consents. Written informed consent was obtained from the blood donors for the use of samples in this research.

The human blood was supplied by the Banc de Sang i Teixits (http://www.bancsang.net/) (Barcelona, Spain). The blood provision from Banc de Sang i Teixits was approved by the Clinical Research Ethics Committee of the Vall d’Hebron Hospital in Barcelona, Spain. The malaria project research was approved by the Ethics Research Committee of the Universidad Autónoma de Madrid, Spain.

### Parasites and cultures

The 3D7A (MR4, MRA-151) and NF54 (kindly provided by Robert E. Sinden) *P*. *falciparum* strains were cultured using a method modified from that of Trager and Jensen [28], in a 5% CO_2_, 5% O_2_ atmosphere at 37°C [29].

### Gametocytogenesis and purification of the mature gametocytes

Purified stage IV-V gametocytes were obtained as previously described [10]. Single point ATP bioluminescence assay and dose–response of positive hits was determined in accordance with the previously published protocol [10].

### Compounds management

Compounds were dispensed using the Echo-CRS system. For the determination of single point activity, 250 or 50 nL from a 1 mM mother solution for 5 and 1 μM final concentration, respectively, were dispensed into the 384-well Greiner plate (ref. 781091), using the HTS GSK layout with 250 or 50 nL DMSO in column 6 (vehicle control) and 250 nL of 100 μM epoxomicin (background control, 0.5 μM final concentration) in column 18. For the dose–response analysis (determination of IC_50_), each row of the 384-well plate contained the serial 1/2 dilutions of two compounds (11 dilutions): the first one interrupted in column 6 and the second one in column 18 (vehicle and background controls, respectively). The plates were kept frozen until use.

### Gametocytocidal assay

Once purified, gametocytes were counted using a Neubauer chamber, and the density was adjusted to 2.5 x 10^5^ gametocytes/mL. Fifty μL of the gametocyte suspension (1.25 x 10^4^ gametocytes) were added to each well of the 384-well plate containing the compounds, using a Multidrop combi (Thermo Electron Corporation). Plates were shaken for 5 sec to homogenize and then incubated at 37°C for 48 h in an atmosphere of 5% CO_2_, 5% O_2_, 90% N_2_. The ATP level of each well was determined using the BacTiter-Glo reagent (Promega) according to the recommendations of the manufacturer. The BacTiter-Glo assay generates a ‘glow-type’ luminescent signal produced by the luciferase reaction, which consists of mono-oxygenation of luciferin catalyzed by luciferase in the presence of Mg^2+^, ATP and molecular oxygen. The luminescence was measured using a plate reader (Victor V, Perkin Elmer). The background value of the assay was obtained as the average ATP level of 16 samples exposed to 1000-fold the IC_50_ of epoxomicin (0.5 μM). The background value was subtracted from every other value of the assay prior to further calculation. The average of 16 replicates of vehicle control (0.5% or 0.1% DMSO) represents 100% of signal. Related to this value, each test concentration was calculated as percentage of control. All the calculations were done using MS Excel software. The compounds which presented inhibition ≥ 90% at 5 μM were progressed to the assay at 1 μM. All the compounds active in the 1 μM single point assay (inhibition ≥ 50%) were tested to determine the exact IC_50_ value (concentration of compound which corresponds to 50% inhibition). These values were obtained from the dose–response curves plotted using Graph Pad software (version 6).

### Interference assay

To discard possible false-positive results, we performed an interference assay in parallel. This evaluated the inhibition of the luciferase reaction by the compounds at a concentration of 50 μM, which is much higher than the maximum concentration tested in the screening assay (5 μM). Tests were done at a final concentration of ATP of 37.5 nM. BacTiter-Glo reagent (Promega) was added and luminescence was measured. The ATP solution was made by dissolving ATP powder (ATP standard from the ATPlite 1 step assay kit, reference 6016731, Perkin Elmer) in culture medium. The positive control used was the Luciferase Inhibitor I Calbiochem (Merck, 119113) at 100 μM.

### Cytotoxicity

Recognition of drug-induced hepatotoxic potential early in the drug development cascade creates opportunities for ranking and prioritizing compounds with lower toxicity [30].

#### Cell line

HepG2, a human Caucasian hepatocellular carcinoma, was supplied by ECACC (ref. 85011430).

#### Routine culture

Cells were grown and maintained in EMEM (Sigma-Aldrich) supplemented with 2mM L-glutamine (Sigma-Aldrich) and 10% fetal calf serum (Perbio). Cultures were maintained at 37°C in a humidified incubator containing 5% CO_2_ and 95% air. Passages were routinely made upon reaching 80–90% confluence. For cytotoxicity experiments, cells were seeded onto 96-well clear bottom black plates coated with type I collagen (Biocoat, Becton Dickinson) at a cell density of 20,000 cells/well.

#### Measurement of cytotoxicity

To determine cytotoxic effects, represented by the Tox_50_ value (the concentration of drug that reduces cell viability by 50%), cells were exposed to serial dilutions of compounds for 48 h at 37°C. The assay medium was as previously described, but supplemented with 5% fetal calf serum instead of 10%. Following the 48 h drug exposure period, the intracellular ATP content was determined using the ATPlite 1 step kit (Perkin Elmer, 6016731). In the presence of ATP, luminescence is emitted as the reaction described earlier for the BacTiter-Glo kit. Percentages of inhibition were calculated relative to vehicle control wells.

### Similarity-based computational search of analogs (CSA)

This chemical analytical method first comprised clustering of the 56 drug-like hits prioritized from the HTS into 39 clusters by using the Complete Linkage algorithm implemented in the Scaffold Hunter program [31]. These clusters were complemented with all the analogs in TCAMS showing a Tanimoto similarity ≥ 0.8 to any of the 56 prioritized compounds (molecules were represented by topological fingerprints in the RDKit cheminformatic toolkit [32]). These expanded clusters were characterized in terms of evidence of gametocyte activity in the neighborhood of the prioritized hits by calculating for each of them the hypergeometric probability *p(m*,*n)* of having *m* active compounds (> 50% inhibition at 5 μM) within the *n* members of the cluster. Larger clusters with an ‘unusually’ high proportion of actives are improbable and, therefore, will have a low value of *p*. These clusters show the largest opportunity for chemical expansion of the prioritized hits within TCAMS.

### Substructure-based medicinal chemistry scaffold indexing (MCSI)

Substructure-based medicinal chemistry scaffold indexing was carried out to facilitate structure–activity relationship studies for prioritized hits from the HTS campaign. This method used as starting points the 39 clusters obtained with the CSA method as well as the corresponding analogs identified for these clusters. For each cluster and its corresponding analogs, an initial proposed scaffold was optimized to yield the minimum common chemical structure shared by the highest number of compounds included in TCAMS. The optimization step was done by adding or subtracting small structural fragments from the previous proposed scaffolds until the optimized scaffold was present in the structure of the highest number of compounds included in TCAMS. In a further step, analysis of the results obtained for these compounds in the initial screen (whole set of compounds tested at a single concentration, 5 μM) (see S1 Table) led to the identification of ‘active’ TCAMS analogs, defined as those compounds not only displaying the defined chemical scaffold but also showing > 50% inhibition in the primary screening campaign, and therefore expected to present an IC_50_ in the low single-digit micromolar range.

### Exflagellation assay

The exflagellation assay was performed as described by Delves et al. [33], with some modifications. Briefly, 200 μL samples of mature *P*. *falciparum* NF54 gametocytes culture in the presence of the corresponding test compound were incubated at 37°C for 48 h in an atmosphere of 5% CO_2_, 5% O_2_, 90% N_2_ in 1.5 mL microfuge tubes. After the incubation period, cells were settled down and medium was carefully removed. The cell pellets were resuspended in 15 μl of complete ookinete medium (RPMI medium with 25 mM HEPES, 50 mg/L hypoxanthine, 2 g/L sodium bicarbonate, 100 μM xanthurenic acid, 20% human serum) pre-warmed at 37°C. Once parasites were resuspended in ookinete medium, they were introduced in a chamber of a FastRead disposable hemocytometer slide and placed on a horizontal surface at room temperature (21°C), to allow cells to homogeneously settle, forming a tight monolayer. After 14 min in the chamber, exflagellation was observed at 10X magnification, and the number of exflagellation centers per field was recorded.

### Standard membrane feeding assay (SMFA)

Gametocyte cultures of the *P*. *falciparum* NF54 strain, kindly provided by Robert Sinden (Imperial College London), were induced at 0.5% asexual parasitemia and 4% hematocrit (RBCs from the Spanish Red Cross) at 40 mL final volume in T75 flasks. Gametocyte production media comprised RPMI 1640 (Sigma, #R5886), 30 mM bicarbonate (Sigma-Aldrich, #S5761) and 5 mM hypoxanthine (Gibco, #11067–030). Media were made complete with 5% human serum A^+^ (Interstate Blood Bank, USA) and 5% AlbuMAX II (Gibco, #11021–037). Cultures were maintained in a gassed incubator at 37°C with 5% CO_2_, 5% O_2_, 90% N_2_ for 14–17 days post-induction, with daily medium replacement without the addition of fresh RBCs. Percentages of mature gametocytes were assessed by microscopic counts (Leica, DM2000) of Giemsa-stained thin blood smears, and functional microgametocytes were assessed for exflagellation activity at room temperature. To test exflagellation, mature gametocyte cultures were centrifuged at 1800 rpm for one minute. The infected RBC pellet was gently mixed with an equal volume of pre-warmed human serum and introduced onto a slide with a coverslip, followed by incubation at room temperature for 10–15 min. The average exflagellation centers per field at 10X magnification was determined by the exflagellation assay described above [33]. Gametocyte counts and exflagellation assays were performed both before and after gametocyte drug treatment (at the time of SMFA).

Two days before gametocyte treatment with compound, spent media was removed and fresh Gametocyte Treatment Medium consisting of RPMI1640 with L-glutamine and 25 mM HEPES (Gibco, #13018–031), 10 mM glucose (Sigma-Aldrich, #G8270), 20 mM bicarbonate (Sigma-Aldrich, #S5761) and 5 mM hypoxanthine (Gibco, #11067) was added. Media were made complete with 10% of human A^+^ serum (Interstate Blood Bank). Five ml of mature gametocyte culture at day 15 post-induction was transferred into each well of a pre-warmed 6-well plate. The selected TCAMS compounds were prepared fresh as a stock solution of 10 mM in 100% of DMSO and added to the mature gametocytes at the concentrations described in Fig 7. Gametocytes were exposed to the compound for a total duration of 48 h. At 24 h post-drug exposure, a fixed volume (3 mL) of spent medium was removed and fresh medium was re-added, accompanied by compound replenishment to obtain the required final concentration. Untreated gametocytes with the same final concentration of DMSO (0.1%) as in treated gametocytes were processed in parallel.

On the day of the feed, cultures were centrifuged at 2500 g for 3 min at 37°C, diluted 1:1 with 100% packed cell volume of fresh RBCs, and finally formulated as artificial mosquito blood meals at 50% hematocrit with pre-warmed human serum. All steps were performed at 37°C. Prepared blood meals were fed in duplicate to 4–6 days old female *An*. *stephensi* mosquitoes for 30–40 min via Parafilm membrane attached to glass feeders (Fisher Scientific, #12831283) connected to a 37°C circulating water bath. Fed mosquitoes were maintained in an incubator at 27°C and 75% relative humidity with 12 h light/dark cycles. At 7–8 days post-feeding, mosquitoes with fully developed ovaries were dissected for midguts (Leica, M80) and incubated in 0.2% mercurochrome solution in distilled water for 10–15 min. The total number of oocysts in individual midguts was counted using a light microscope (Leica, DM2000) under a 10X objective (100X magnification). Both infection prevalence (percentage of mosquitoes with one or more oocysts) and mean oocyst intensity of infection were defined in each treatment. The percentage block in transmission and the percentage reduction in mean oocyst intensity were calculated after normalizing to the Control DMSO-treated sample.

The Mann-Whitney U-test was used to compare the statistical significance between the different treatments and the control.

## Supporting Information

S1 Table Percent inhibition of *P*. *falciparum* gametocytes by TCAMS compounds at a 5 μM concentration. (XLSX)

S2 Table List of 373 compounds which decreased ATP levels in *P*. *falciparum* gameotcytes by ≥ 50% at a 1 μM concentration.sequence (XLSX)

S3 Table An interference assay, using a panel of 167 compounds tested at 50 μM, showed no significant inhibition of the BacTiter-Glo reaction. (XLSX)

S4 Table Correlation between IC_50_ determined against *P*. *falciparum* gametocytes and against asexual stages for 373 compounds with ≥ 50% inhibition of *P*. *falciparum* gametocytes at a 1 μM concentration. (XLSX)

S5 Table List of 98 compounds displaying anti-gametocyte potencies < 2 μM and a selectivity ratio > 10. (XLSX)

S6 Table Physicochemical properties of 98 selected compounds with anti-gametocyte potencies < 2 μM and a selectivity ratio > 10. (XLSX)

S7 Table Biological profile of the 56 prioritized compounds. (XLSX)

S8 Table Chemical analysis of the 56 prioritized hits by CSA and MCSI methods. (XLSX)

S9 Table Similarity-based computational analysis identified 434 analogs associated to 25 clusters and 14 singletons. (XLSM)

S10 Table Substructure-based medicinal chemistry scaffold indexing identified 1252 analogs associated to 36 clusters and 6 singletons. (XLSM)
